# The Safe Path at the Fork: Ensuring Replication-Associated DNA Double-Strand Breaks are Repaired by Homologous Recombination

**DOI:** 10.3389/fgene.2021.748033

**Published:** 2021-09-27

**Authors:** Jac A. Nickoloff, Neelam Sharma, Lynn Taylor, Sage J. Allen, Robert Hromas

**Affiliations:** ^1^ Department of Environmental and Radiological Health Sciences, Colorado State University, Ft. Collins, CO, United States; ^2^ Division of Hematology and Medical Oncology, Department of Medicine and the Mays Cancer Center, University of Texas Health Science Center, San Antonio, TX, United States

**Keywords:** genome instability, DNA damage, DNA double-strand breaks, structure-specific nucleases, replication stress

## Abstract

Cells must replicate and segregate their DNA to daughter cells accurately to maintain genome stability and prevent cancer. DNA replication is usually fast and accurate, with intrinsic (proofreading) and extrinsic (mismatch repair) error-correction systems. However, replication forks slow or stop when they encounter DNA lesions, natural pause sites, and difficult-to-replicate sequences, or when cells are treated with DNA polymerase inhibitors or hydroxyurea, which depletes nucleotide pools. These challenges are termed replication stress, to which cells respond by activating DNA damage response signaling pathways that delay cell cycle progression, stimulate repair and replication fork restart, or induce apoptosis. Stressed forks are managed by rescue from adjacent forks, repriming, translesion synthesis, template switching, and fork reversal which produces a single-ended double-strand break (seDSB). Stressed forks also collapse to seDSBs when they encounter single-strand nicks or are cleaved by structure-specific nucleases. Reversed and cleaved forks can be restarted by homologous recombination (HR), but seDSBs pose risks of mis-rejoining by non-homologous end-joining (NHEJ) to other DSBs, causing genome rearrangements. HR requires resection of broken ends to create 3’ single-stranded DNA for RAD51 recombinase loading, and resected ends are refractory to repair by NHEJ. This Mini Review highlights mechanisms that help maintain genome stability by promoting resection of seDSBs and accurate fork restart by HR.

## Introduction

Cells maintain relatively stable genomes during cell division to prevent accumulation of potentially oncogenic mutations. Cells proliferate despite copious DNA damage caused by endogenous and exogenous agents. Endogenous agents include reactive oxygen species (ROS) from oxidative metabolism, nucleases and other enzymes such as members of the AID/APOBEC DNA deaminase family, mis-incorporated ribonucleotides, and DNA chemical lability (Gates, 2009; Ciccia and Elledge, 2010; Nick Mcelhinny et al., 2010; Williams et al., 2013; Petljak and Maciejowski, 2020; Juan et al., 2021). DNA damage is induced directly or indirectly by exogenous chemical agents including alkylating agents and other DNA-reactive chemicals including cancer chemotherapeutics, and pollutants in food, water and air. Physical agents that damage DNA include ultraviolet light and ionizing radiation (Friedberg et al., 2014; Nickoloff et al., 2020a). DNA damage comprises chemical changes to bases and the sugar-phosphate backbone, base loss, single-strand breaks, double-strand breaks (DSBs), and intra- and interstrand crosslinks. Protein-DNA crosslinks arise when topoisomerases are trapped in covalent linkages to DNA by topoisomerase poisons, commonly used in cancer therapy (Pommier et al., 2006; Deweese and Osheroff, 2009; Friedberg et al., 2014; Thomas and Pommier, 2019; Riccio et al., 2020). DNA damage detection, signaling and repair systems evolved to manage these threats, termed the DNA damage response (DDR). Nearly all DNA lesions block replicative polymerases (Pol ε, Pol δ), causing fork stalling and fork collapse, and cells manage this replication stress by activating S phase-specific DDR pathways. Replication stress is also caused by depletion of nucleotide pools by hydroxyurea, and DNA polymerase inhibitors (Vesela et al., 2017).

Unstressed cells suffer > 100,000 DNA lesions per day, with a steady state of ∼10,000 lesions per cell (Tubbs and Nussenzweig, 2017). Thus, human cells manage an average of ∼2000 DNA lesions per day in each chromosome, or roughly one lesion per 30 kbp per day. Given that typical human replicons are 75–175 kbp (ranging from 30–450 kbp) (Ligasova et al., 2009) and S phase comprises ∼30% of a 24 h cell cycle, each active replicon will harbor > 2 DNA lesions (assuming lesions arise at similar rates throughout the cell cycle). The DNA replication machinery faces many other challenges in addition to DNA damage. The replisome helicase complex (CDC45, MCM2-7 and GINS), or more often replicative polymerases, slow or stall at unusual structures such as G-rich sequences that form G-quadraplexes, common fragile sites, and hairpins at inverted repeats and CAG/CTG triplet repeats (Bochman et al., 2012; Barlow et al., 2013; Zeman and Cimprich, 2014; Gadaleta and Noguchi, 2017; Kaushal and Freudenreich, 2019; Spiegel et al., 2020; Poggi and Richard, 2021). Replication stress is also caused by conflicts with R-loops formed during transcription, particularly at fragile sites, telomeres, and ribosomal DNA, and by proteins that bind tightly to DNA (Ivessa et al., 2003; Bermejo et al., 2012; Kotsantis et al., 2016; Billard and Poncet, 2019; Gomez-Gonzalez and Aguilera, 2019). These so-called ‘difficult-to-replicate’ sequences are encountered by replisomes in every S phase. Activated oncogenes in cancer cells also increase replication stress through de-regulated replication origin firing (Hills and Diffley, 2014). Persistent fork stalling can cause replisome dissociation, or forks may be cleaved by nucleases to yield seDSBs, both of which have been termed ‘fork collapse’ (Cortez, 2015). We discuss the distinct challenges associated with repair of frank, two-ended DSBs vs replication-associated seDSBs, and recent studies that illuminate mechanisms that ensure accurate, timely repair and restart of stressed replication forks.

### Double-Strand Break Repair: A Double-Edged Sword

**FIGURE 1:**
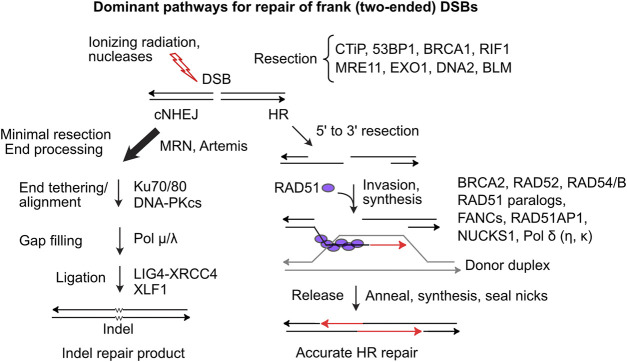
Dominant two-ended DSB repair pathways. **(Left)** cNHEJ is the dominant pathway for repairing two-ended DSBs. cNHEJ acts on blunt or minimally processed ends bound by Ku70/Ku80 and DNA-PKcs. Short gaps are filled and ends are ligated to complete repair, typically with small insertions or deletions at the repair junction **(Right)** HR initiates with 5′-3′ resection and binding of ssDNA by RPA, which is replaced by RAD51 in a reaction mediated by BRCA2 and RAD51 paralogs. The RAD51 nucleoprotein filament invades homologous duplex DNA, assisted by RAD54, RAD54B and other factors. RAD51 dissociates and the invading end is extended and then released allowing pairing to ssDNA on the opposite side of the DSB. Gaps are filled and ends ligated to complete accurate DSB repair.

DSBs are dangerous DNA lesions that can cause genome instability and cell death. These threats are mitigated by several DSB repair pathways with different levels of accuracy. In mammalian cells, classical non-homologous end-joining (cNHEJ) is the dominant DSB repair pathway (Figure 1). An early cNHEJ step involves Ku70/Ku80 binding to broken ends. Ku protects ends from degradation and recruits DNA-PKcs, activating the DNA-PK holoenzyme, which promotes end-alignment and rejoining by LIG4-XRCC4 and other factors (Chang et al., 2017). cNHEJ operates on blunt or short overhanging ends and is error-prone, typically producing small insertion/deletion (indel) mutations that may be deleterious, but in certain contexts are quite beneficial, as in the generation of diverse antibody receptor genes (Arya and Bassing, 2017). Alternative NHEJ (aNHEJ) is a backup NHEJ pathway mediated by DNA ligase III when ends anneal at microhomologies. aNHEJ is more error-prone than cNHEJ, producing larger deletion mutations and translocations (Simsek et al., 2011; Iliakis et al., 2015; Sallmyr and Tomkinson, 2018). HR repair of DSBs is generally error-free as it employs an undamaged homologous sequence as repair template. HR initiates when broken ends are resected by > 50 nt, exposing long 3’ ssDNA extensions initially bound by RPA that is exchanged with RAD51 which catalyzes strand invasion into homologous duplex DNA (Figure 1). End resection is regulated and mediated by many factors. CtIP, phosphorylated by CDK, ATM and ATR (limiting resection to S/G2 phases), activates MRE11 nuclease (in complex with RAD50-NBS1) to effect limited end resection (Anand et al., 2016). BRCA1-BARD1 promotes end resection in part by ubiquitination of H2A and by blocking the anti-resection factors 53BP1-RIF1/Shieldin (Mirman et al., 2018; Densham and Morris, 2019). Extensive resection is effected by EXO1 and by DNA2-BLM (Zhao et al., 2020).

Limiting HR largely to S/G2 phases promotes sister chromatid use as HR templates. RAD51 loading and strand invasion are mediated by many factors including BRCA1, BRCA2, five RAD51 paralogs (RAD51B/C/D, XRCC2/3), members of the Fanconi’s anemia (FANC) protein family, RAD54/B, and RAD51AP1 and its paralog NUCKS1 (Pires et al., 2017; Wright et al., 2018; Niraj et al., 2019; Maranon et al., 2020). After repair synthesis, the extended strand reanneals with the resected end on the other side of the DSB, and gaps are filled and ligated to complete repair. Alternative HR pathways yield double Holliday junctions that can be resolved with or without crossovers (Piazza and Heyer, 2019). Single-strand annealing (SSA) is a RAD51-independent HR pathway that requires RAD52 and is observed, for example, in BRCA2-mutated breast cancer cells (Tutt et al., 2001). SSA is error-prone as resected ends anneal at complementary sequences, either between repeats flanking a DSB deleting one repeat and intervening sequences, or between repeats on different chromosomes causing translocations (Weinstock et al., 2006; Nickoloff et al., 2008; Bhargava et al., 2016). Failure to repair DSBs can cause chromosome loss and cell death, but it’s clear that DSB repair also poses significant risks to genome integrity, including genome rearrangements, lethal dicentric chromosomes, and bridge-breakage-fusion cycles (Fenech et al., 2011; Murnane, 2012; Feijoo et al., 2014).

### DNA Damage and Replication Stress Responses

The DDR elicits checkpoint responses that arrest or slow cell cycle progression and stimulate DNA repair. Checkpoints arrest or slow cell cycle progression at the G1/S transition, within S phase (intra-S checkpoint) and the G2/M transition. The DDR also promotes programmed cell death if damage is excessive (Roos and Kaina, 2013; Tian et al., 2015; Mladenov et al., 2016; Blackford and Jackson, 2017). Cells initially respond to replication stress by protecting stalled forks and maintaining replisomes until the stress is resolved, otherwise various mechanisms are employed to restart or rescue the fork to ensure timely completion of DNA replication before mitosis. The DDR is important because defects in DDR proteins typically cause genome instability that can drive carcinogenesis (Tubbs and Nussenzweig, 2017), and because DDR proteins are important targets to augment cancer therapy (Nickoloff et al., 2017; Desai et al., 2018; Pilie et al., 2019; Nickoloff et al., 2020b; Baillie and Stirling, 2021). Central to the DDR are three members of the phosphatidyl inositol 3′ kinase-related kinase (PIKK) family, DNA-PKcs, ATM, and ATR, all of which play important roles in DSB repair. These PIKKs are structurally related and phosphorylate target proteins at canonical serine/threonine-glutamine (S/T-Q) sites, as well as non-canonical sites. PIKKs auto- and cross-phosphorylate each other and other DDR proteins to regulate checkpoints and repair (Liu et al., 2012; Marechal and Zou, 2013; Ashley et al., 2014; Boohaker and Xu, 2014; Blackford and Jackson, 2017). Although PIKKs phosphorylate overlapping targets, they have distinct roles in specific DSB repair contexts.

DNA-PKcs plays a critical role in cNHEJ repair of two-ended DSBs induced, for example, by ionizing radiation or nucleases (Chang et al., 2017). The catalytic subunit of DNA-dependent protein kinase, DNA-PKcs, is activated when complexed with Ku70/Ku80-bound DSB ends. DNA-PKcs mediates cNHEJ and checkpoint responses by phosphorylating itself, Ku, MRE11, RAD50, XRCC4-6, XLF, artemis, and histone H2AX, as well as proteins involved in transcription, cell growth, heat shock responses, and viral DNA integration (Anisenko et al., 2020). ATM (ataxia telangiectasia mutated) is also activated by DSBs, mediated by the MRE11-RAD50-NBS1 complex. ATM promotes frank DSB repair by HR, phosphorylating hundreds of targets including itself, BRCA1, NBS1, H2AX, p53, MDC1, and Chk2 kinase (Blackford and Jackson, 2017). Phosphorylated/activated Chk2 phosphorylates effector proteins that mediate HR and cell cycle arrest (among other processes), including BRCA1, BRCA2, p53, CDC25A, and RB (Marechal and Zou, 2013; Zannini et al., 2014). ATM autophosphorylation promotes ATM binding to MDC1 which binds to phospho-S139 S H2AX (γ-H2AX), and promotes spreading of the *γ*-H2AX signal to Mbp chromatin domains flanking DSBs (Savic et al., 2009). ATR plays a central role in replication stress responses (Yazinski and Zou, 2016), and is activated by ssDNA formed when blocked DNA polymerase decouples from the helicase and DNA unwinding continues ahead of the fork (Cortez, 2005). ATR is activated by a multi-step process that involves ATR-ATRIP recruitment to RPA-bound ssDNA, RAD17-RFC, Claspin, TopBP1, and 9-1-1 (Yazinski and Zou, 2016). ATR is also activated by an NBS1-dependent mechanism (Shiotani et al., 2013). Recently, ATR activation was shown to be mediated by the RPA-binding factor ETAA1 in an TopBP1-independent manner; dual inactivation of ETAA1 and TopBP1 abrogates ATR signaling and is synthetically lethal (Haahr et al., 2016). Activated ATR phosphorylates Chk1 which slows cell cycle progression in S/G2 phases and delays late origin firing (Yazinski and Zou, 2016).

All three PIKKs respond to DSBs and phosphorylate residues in RPA (Anantha et al., 2007; Oakley and Patrick, 2010; Ashley et al., 2014), yet they also have kinase-independent DDR roles. For example, distinct phenotypes result from DNA-PKcs null mutations vs kinase genetic inactivation or drug inhibition (Allen et al., 2003; Shrivastav et al., 2009; Menolfi and Zha, 2020), and cells lacking DNA-PKcs compensate by downregulating ATM expression (Peng et al., 2005; Shrivastav et al., 2009). Despite this crosstalk, current evidence indicates that DNA-PKcs promotes frank DSB repair by cNHEJ, ATM promotes frank DSB repair by HR, and ATR promotes repair of replication-associated DSBs by HR (Blackford and Jackson, 2017; Pilie et al., 2019).

### Restarting Stalled and Collapsed Replication Forks

**FIGURE 2:**
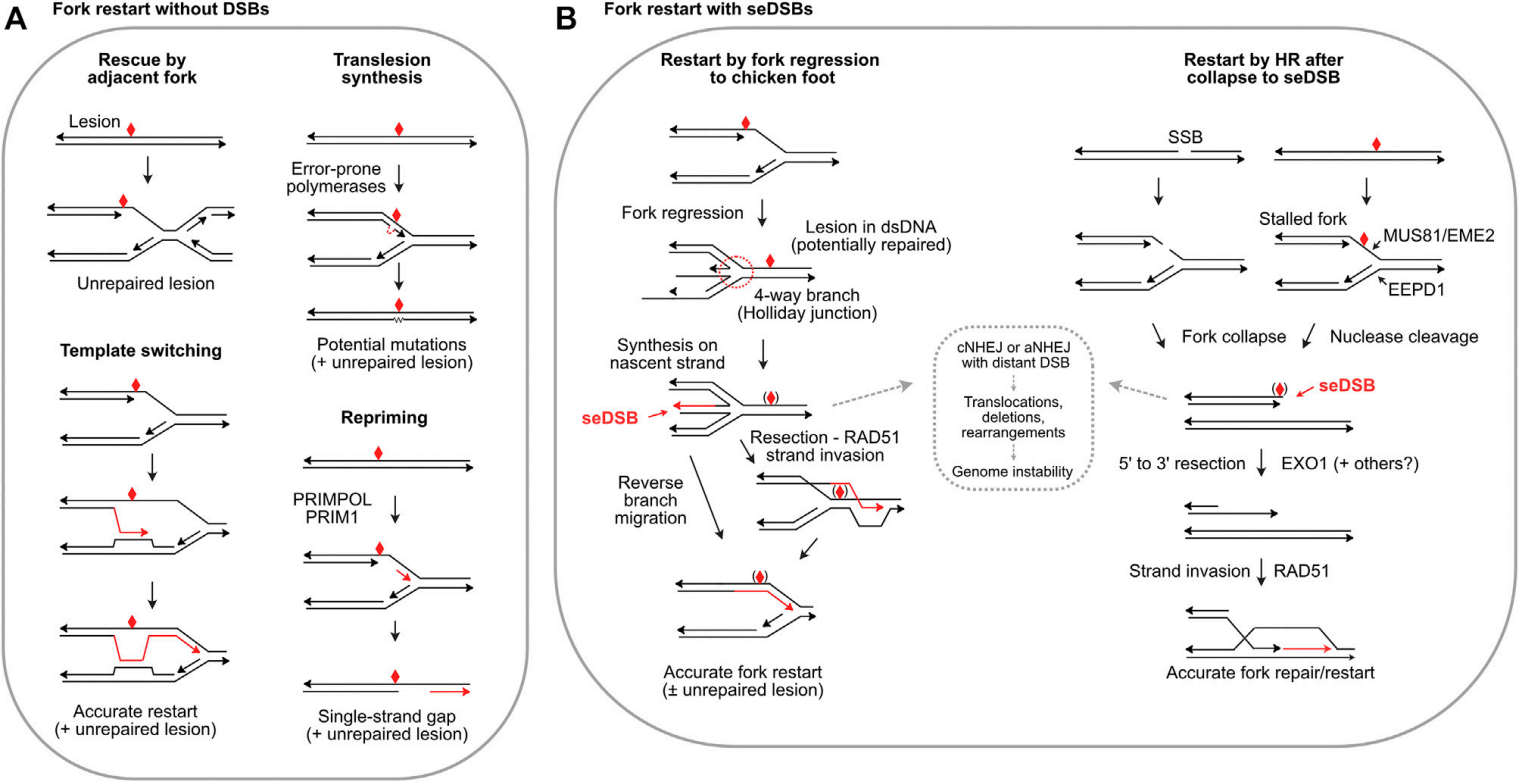
Fork restart mechanisms. **(A)** Fork restart mechanisms that do not create seDSBs. Illustrated are rescue by an adjacent fork, TLS, template switching, and repriming. Blocking lesions are shown by red symbols and repair or bypass synthesis is shown by red arrows. **(B)** Fork restart by fork regression, fork encounters with a single-strand break (SSB), or fork cleavage, which create seDSBs. Regressed forks allow synthesis past the blocking lesion using the nascent strand as template. Reverse branch migration restarts the fork, or RAD51 may load onto a resected end allowing strand invasion downstream of the blocking lesion. Blocking lesions may be bypassed or repaired, indicated by symbols in parentheses. Collapsed forks due to encounter with single-strand breaks or fork cleavage can restart by RAD51-mediated strand invasion, i.e., break-induced replication. The strand invasion restart pathways are mediated by HR; fork regression/reversal is not an HR pathway, but RAD51 is still required to protect the nascent strands in the chicken foot. HR defects and HR inhibitors may shunt seDSB intermediates to cNHEJ or aNHEJ (dashed box) causing genome instability.

Given the central importance of DNA replication, it is not surprising multiple replication stress response mechanisms evolved. The initial response to replication stress is to stabilize replisomes at stalled forks to prevent fork collapse (Tye et al., 2021). Stalled forks often reverse to a so-called ‘chicken foot’ structure wherein nascent strands anneal, producing a four-way junction resembling a Holliday junction, but with a seDSB (Figure 2B). Fork protection involves the end resection inhibitor RIF1 (Mukherjee et al., 2019); MRNIP (Bennett et al., 2020); the de-ubiquitinating enzyme USP1 which suppresses translesion synthesis (TLS) by regulating PCNA (Lim et al., 2018); HR proteins RAD51, BRCA1, BRCA2 and FANCD2 (Schlacher et al., 2012; Rickman and Smogorzewska, 2019; Rickman et al., 2020); and the RAD51 regulator RADX (Bhat et al., 2018). Fork protection defects generally increase cytotoxicity of replication stress agents and are thus targets to augment cancer therapy. Fork protection can provide sufficient time to repair blocking lesions, but if not resolved in timely manner, adjacent forks may rescue stressed forks (Figure 2A), passively or through checkpoint activation of an adjacent dormant origin (Yekezare et al., 2013; Brambati et al., 2018).

Several fork restart mechanisms do not repair the blocking lesion, and thus are damage tolerance pathways. TLS involves transient replacement of replicative DNA polymerases with error-prone, TLS polymerases including Pol β, κ, η, τ, and ζ, and Rev1 (Goodman and Woodgate, 2013; Ma et al., 2020) (Figure 2A). Repriming by PRIMPOL and PRIM1 restarts replication downstream of blocking lesions, bypassing lesions and leaving single-strand gaps in nascent DNA (Quinet et al., 2021) (Figure 2A). Template switching uses sister chromatids to bypass blocking lesions and is generally error-free (Figure 2A), but poses risks of genome rearrangement from replisome switching to non-sister templates (Lehmann et al., 2020). Two additional restart pathways are fork reversal to a Holliday junction-like structure followed by fork restoration, and fork cleavage by structure-specific nucleases including the 3′ nuclease MUS81 (with EME2) (Pepe and West, 2014) and the 5’ nuclease EEPD1 (Wu et al., 2015; Sharma et al., 2020) (Figure 2B). Metnase is a structure-specific nuclease that promotes fork restart, but Metnase does not cleave stalled forks and instead may process flaps that arise later (Sharma et al., 2020). SLX1-SLX4 is another structure-specific nuclease that resolves branched replication intermediates, and although it cleaves many types of branched structures including replication fork structures *in vitro*, direct evidence that it cleaves stalled forks *in vivo* is lacking (Falquet and Rass, 2019; Xu et al., 2021). seDSBs at cleaved forks can be repaired and the fork re-established/restarted accurately if the end is resected and invades the sister chromatid (Figure 2B), often termed break-induced replication (BIR). Repair of collapsed forks by BIR may function primarily during S or G2 phases to ensure complete DNA replication prior to mitosis, but recent studies show that BIR also operates during mitotic DNA synthesis (MiDAS), an important mechanism for completing replication in common fragile sites, telomeres, and other under-replicated DNA during mitosis (Epum and Haber, 2021).

### Minimizing Risks Associated With Replication Stress

Each fork rescue pathway poses risks: TLS is error-prone, producing point mutations, repriming yields vulnerable single-strand gaps, template switching poses risks of genome rearrangements (Figure 2A), and fork reversal/cleavage creates seDSBs which pose risks of aberrant cNHEJ causing genome rearrangements (Figure 2B). Interestingly, cNHEJ factors are present at seDSBs; similar to their presence at telomeres, cNHEJ factors at seDSBs may protect ends but further cNHEJ steps are suppressed (Sui et al., 2020; Audoynaud et al., 2021). Rescue by an adjacent fork might seem the least risky pathway: simply waiting for rescue by an adjacent fork (or repair of blocking lesion) would eliminate risks posed by other pathways. However, cells tightly regulate replication timing (and cell cycle progression), especially during embryonic development. The importance of timely fork restart is illustrated by increased genome instability, developmental defects, and cell death when fork restart is delayed by as little as 10 min (Kim et al., 2014; Kim et al., 2015; Wu et al., 2015; Chun et al., 2016). Timely fork restart probably limits the formation of toxic (cell lethal) recombination intermediates, thought to be unresolvable branched structures. In yeast, HR proteins and helicases drive formation of toxic recombination intermediates, and they are prevented or resolved by several factors including Srs2, Sgs1-Top3, Smc5/6, *Mus*81-Mms4, and Dna2 (Menolfi et al., 2015; Keyamura et al., 2016; Falquet et al., 2020). For example, toxic recombination intermediates cause synthetic lethality in *sgs1*Δ *mus81*Δ double mutants, but viability is restored by defects in HR genes including *RAD51*, *RAD52*, *RAD54*, *RAD55* and *RAD57* (Gangloff et al., 2000; Fabre et al., 2002; Bastin-Shanower et al., 2003).

Although it remains unclear how cells choose among various lesion bypass and fork restart pathways, it is likely that the types of blocking lesions, and the extent (local vs genome-wide) and duration of replication stress are determining factors. For example, certain blocking lesions may be promptly bypassed by TLS with sufficient accuracy, such as UV-induced T-T dimers (Washington et al., 2000), whereas specific types of lesions, high lesion loads, or persistent replication stress may require potentially riskier choices.

Despite the risks associated with seDSBs generated during fork regression and fork cleavage, these mechanisms are very common, particularly in human cells (Pepe and West, 2014; Zellweger et al., 2015; Meng and Zhao, 2017). The accuracy of HR-mediated fork restart may outweigh risks of error-prone bypass mechanisms like TLS. Given this, how might cells mitigate risks of cNHEJ acting on resulting seDSBs? A recently described mechanism operates at forks stalled by collision with opposing transcription R-loops in which MUS81 cleaves the fork, and the resulting ends are rejoined by LIG4-XRCC4 with assistance by RAD52-mediated strand annealing and PolD3, a non-catalytic subunit of Pol *d* (Chappidi et al., 2020). This pathway does not involve the core cNHEJ factor Ku, and thus prevents joining of the seDSB to other DSBs and genome instability. As noted above, resected DNA ends are poor cNHEJ substrates. As shown in Figure 2B, regressed forks initially have overhanging ends, unless or until nascent strand synthesis creates blunt ends. Thus, the overhanging ends in early fork regression intermediates are intrinsically protected from cNHEJ, and end-protection by RAD51 and other factors appears to reinforce cNHEJ suppression. In addition, ATM promotes dissociation of DNA-PK from seDSBs, suppressing cNHEJ (Britton et al., 2020). In the case of fork cleavage by either MUS81-EME2 or EEPD1 (or fork collapse at nicks), the initial state is a blunt, or nearly blunt seDSB, i.e., an excellent cNHEJ substate. Recent studies have shown that 5’ fork cleavage by EEPD1 is strongly biased toward end-resection because EEPD1 recruits the key resection nuclease EXO1 to cleaved forks (Wu et al., 2015; Kim et al., 2017). EEPD1 first appeared in late chordates/early vertebrates ∼450–670 Mya (Zerbino et al., 2018), and it may have been selected to augment MUS81-mediated fork restart to manage increased replication stress associated with expanding genomes. Metnase evolved even more recently, ∼50 Mya, (Cordaux et al., 2006), and Metnase also recruits EXO1 to cleaved forks (Kim et al., 2016). Thus, EEPD1 and Metnase both promote HR-mediated fork restart by recruiting EXO1 to promote resection, minimizing cNHEJ of seDSBs at cleaved forks. It is unknown whether resection is also promoted during MUS81-mediated fork restart. MUS81 is not known to interact with EXO1, although EXO1 and MRE11 degrade (unprotected) reversed forks that force fork rescue by MUS81 (Lemacon et al., 2017). The expansion of genomes in higher eukaryotes may have created selection pressure for EEPD1/Metnase fork processing nucleases coupled to EXO1, driving a shift toward accurate, HR-mediated fork restart.

## Concluding Remarks

Replication stress is intimately tied to cancer etiology and treatment. Replication stress causes genome instability that drives cancer progression, and it is caused by oncogenic stress and damage induced by genotoxic chemo- and radiotherapeutics. The DDR plays critical roles in managing replication stress, and inhibitors of DDR factors are promising targets to effect tumor-specific cell killing in mono-therapy or as adjuncts to genotoxic therapy (O'Connor, 2015; Pearl et al., 2015; Kirsch, 2018; Pilie et al., 2019; Trenner and Sartori, 2019; Nickoloff et al., 2020b). Several specific and broader questions remain. For example, are forks cleaved by MUS81-EME2 also preferentially resected (by EXO1? by DNA2-BLM?) as with EEPD1-cleaved forks? How do different types of lesions, lesion loads, or the DDR determine choices among stressed fork restart mechanisms? And can we manipulate DDR signaling or structure-specific nucleases to more effectively and selectively kill tumor cells, in monotherapy or by augmenting conventional chemo- or radiotherapy? Clarifying these questions will promote the development more effective targeted cancer therapeutics.
